# Reflections on prevention and treatment of post-thyroidectomy hypoparathyroidism: current management approaches and future prospects

**DOI:** 10.3389/fendo.2026.1768391

**Published:** 2026-02-27

**Authors:** Jia Li, Zilai Hu, Zhiyuan Ma, Guoli Feng, Lanhai Zhang, Suansuan Zhu, Jinyan Hu, Wei Han, Taolang Li

**Affiliations:** 1 Department of Hepatobiliary Pancreatic and Thyroid Surgery, Department of General Surgery, People's Hospital of Zunyi City Bozhou District, Zunyi, China; 2 Department of Thyroid and Breast Surgery, Department of General Surgery, Affiliated Hospital of Zunyi Medical University, Zunyi, China

**Keywords:** management, parathyroid organoid, postoperative hypoparathyroidism, postsurgical hypocalcemia, recombinant PTH, thyroid surgery, treatment

## Abstract

Postoperative hypoparathyroidism (hypoPT) is one of the predominant and severe complications of thyroid surgery. This condition manifests as suboptimal circulatory levels of parathyroid hormone (PTH), engendering multifaceted systemic perturbations. This comprehensive review elucidates the underlying pathophysiological mechanisms and clinical presentations associated with hypoparathyroidism. Moreover, methodologies for the precise recognition and preservation (naked eye, near-infrared autofluorescence, indocyanine green, carbon nanoparticles, and mitoxantrone hydrochloride) of the parathyroid gland during thyroid surgery have been explored, along with recent therapeutic innovations (palopegteriparatide and parathyroid organoids). Finally, this review prospects and interventions via organoids are contemplated. The aim of the literature is to recapitulate the knowledge and the gaps in this field, increasing awareness of postoperative hypoparathyroidism, and improve the prognosis for patients afflicted with hypoparathyroidism.

## 1 Introduction

**Figure 1:**
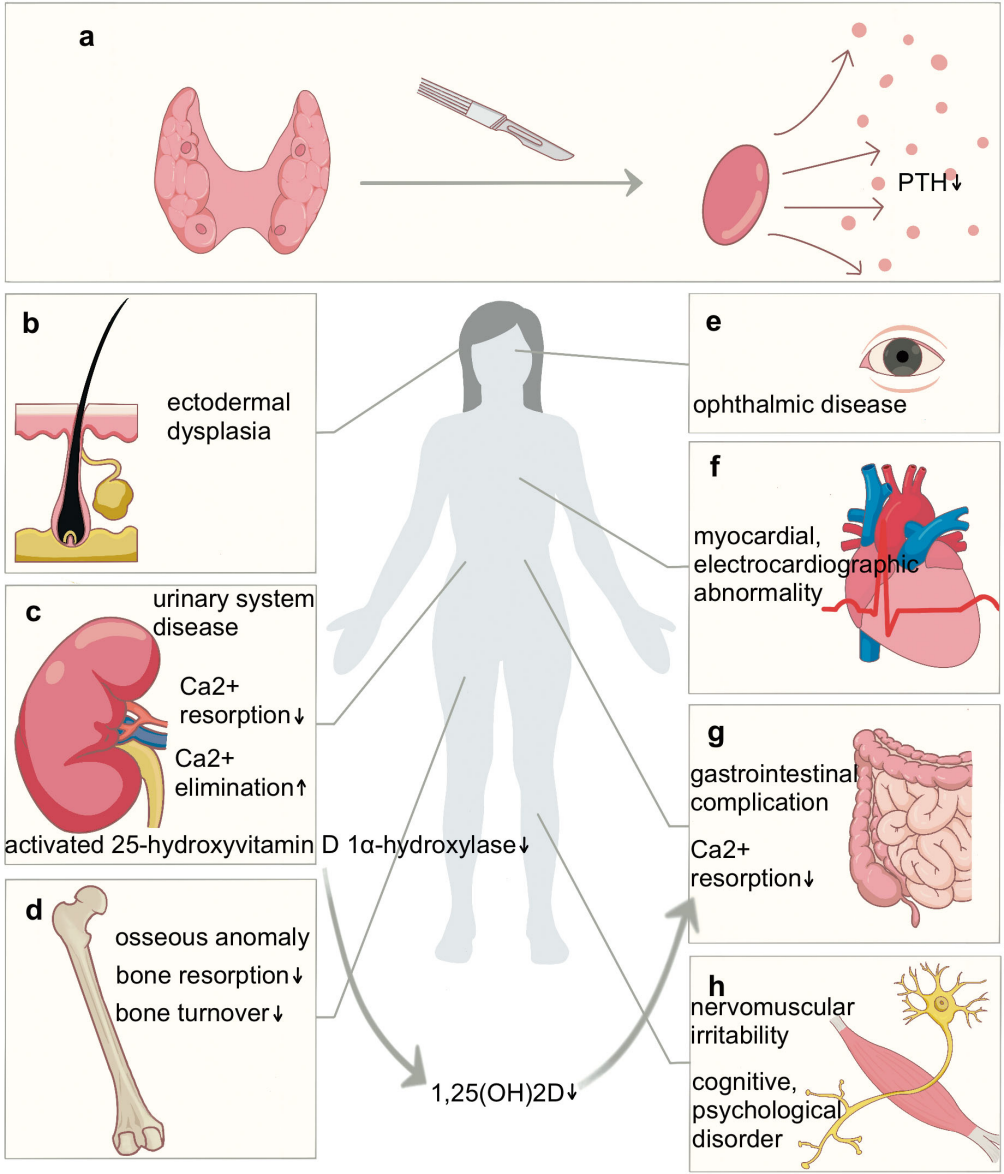
Surgery-induced parathyroid dysfunction resulted in the manifestation of multi-systemic lesions across the body. **(a)** Surgical injury to the parathyroid gland results in insufficient circulating levels of PTH. **(b)** Ectodermal dysplasia presents as rough and dry skin, thinning hair, and alopecia areata. **(c)** Hypercalciuria, urolithiasis, renal calcinosis, chronic renal insufficiency occur and indirectly impact calcium assimilation in the intestines. **(d)** Osseous anomalies include osteosclerosis, cortical thickening, bone pain, skeletal deformities, fractures, and dental hypoplasia. **(e)** Ophthalmologically, cataracts and corneal calcification occur. **(f)** Myocardial and electrocardiographic abnormalities manifest as arrhythmias, prolonged corrected QT intervals, and congestive heart failure. **(g)** The gastrointestinal system is also affected by chronic constipation. **(h)** Neurologically and muscularly, symptoms such as tingling, paralysis around the mouth, a positive Chvostek’s sign and a positive Trousseau’s sign can manifest as irritability. Cognitively and psychologically, patients exhibit fatigue, cerebral lethargy (“brain fog”), affective disturbances, and insomnia. Furthermore, there is a significant increase in the risk of ectopic basal ganglia calcification and fungal infections.

Hypoparathyroidism (hypoPT) is the most prevalent complication after thyroid surgery, and understanding its pathogenesis and existing preventive measures will contribute to reducing the incidence of hypoPT. This is an endocrinological disorder typified by insufficient circulating levels of parathyroid hormone (PTH) (1). PTH is instrumental in maintaining mineral ion dynamic equilibrium. In the circulatory system, PTH interacts primarily with G-protein coupled receptors—PTH1 receptor (PTH1R) (2) and PTH-related protein receptor (PPR) (3). These receptors are highly expressed in renal and osseous tissues, leading to diminished renal calcium elimination (4), the promotion of bone resorption, a heightened bone turnover rate, and regulated bone remodeling (5). Furthermore, the synthesis of the bioactive form of vitamin D (1,25-dihydroxyvitamin D) is contingent upon the role of PTH in catalyzing renal 25-hydroxyvitamin D1α-hydroxylase (6). This enzymatic activity indirectly modulates calcium assimilation in the intestines. PTH deficiency culminates in outcomes such as hypocalcemia, hyperphosphatemia, amplified urinary calcium excretion, increased osteoporotic tendencies, and other associated clinical symptoms (**Figure 1**).

The identification and preservation of parathyroid glands during thyroid surgery, along with comprehensive postoperative treatment measures, have been longstanding focal points for surgeons. Addressing permanent hypoparathyroidism poses a challenge, as there is a lack of completely effective corrective methods with limited treatment-associated complications. The recent development of functional parathyroid organoids has emerged as a notable advancement in this field. The main objective of this study was to elucidate postsurgical hypoPT, summarize the efficacy and constraints of related management strategies, anticipate the latest advancements in research, and ultimately enhance the awareness of postoperative hypoparathyroidism.

## 2 Clinical manifestations

The symptomatic presentation of hypoPT primarily depends on the rate, extent, and persistence of the decrease in the serum calcium concentration. Calcium and phosphate metabolic disruptions induce a variety of systemic or localized clinical indicators.

Acute hypocalcemia often presents as tetany and is occasionally accompanied by symptoms of laryngospasm, stridor, seizures, and the occurrence of epileptiform activity. When it progresses to chronic hypocalcemia could impairs multiple systems in the body (**Figure 1**) (7–9). Conventionally, PTH was considered to affect the central nervous system (CNS) mainly by regulating serum blood calcium, but recent studies have confirmed that PTH can cross relatively permeable areas of the blood-brain-barrier (BBB), thereby exerting direct and complex effects on the brain. Studies have confirmed that abnormal PTH levels are directly associated with neuronal calcium dysregulation, hypoperfusion, and disruption of neuronal signaling (10). PTH1R was detected in multiple critical brain regions, including the hypothalamus, hippocampus, cerebral cortex, cerebellum, astrocyte and choroid plexus, provided evidence of the neuroprotective role of PTH (11). The anxiolytic and anti-depressant effects of PTH may be related to its action on PTH receptors in the hypothalamus-pituitary-adrenal (HPA) axis (12). In an Alzheimer’s disease mouse model, PTH treatment was observed to suppresses astrocyte senescence and expression of proinflammatory cytokines, resulting in a decrease in glial cell activation, dystrophic neurites, and amyloid-beta (Aβ) deposition, and ultimately improving the cognitive function (13). Furthermore, in a model of ischemic stroke, PTH increased the expression of trophic and regenerative factors including vascular endothelial growth factor (VEGF) and brain-derived neurotrophic factor (BDNF), facilitated the migration of neuroblasts from the subventricular zone (SVZ), thereby enhanced the neurovascular regeneration and increased the number of newly generated neurons in the peri-infarct cortex (14). Therefore, we can explore PTH analogs capable of effectively crossing the BBB as potential therapeutic agents for mood disorders. The clinical management of patients with hypoPT should emphasize the assessment of their psychiatric symptoms and consider them as the endpoint.

Consequently, patients with hypoPT complain of neuropsychological and cognitive symptoms and show a low quality of life. Patients possibly need pharmacological regimens and recurrent hospital admissions throughout life. Even after prolonged conventional pharmacological therapy, various targeted organ dysfunctions could trouble patients, such as hypercalciuria, urolithiasis, renal calcinosis, and chronic renal insufficiency. All of these factors, in turn, impose a substantial economic burden on the families of patients and markedly decrease their quality of life (15, 16).

## 3 Classification

According to the guidelines from the American Thyroid Association Surgical Affairs Committee Statement, hypoPT is classified into distinct categories and can be defined by the following diagnostic metrics: biochemical, clinical, and relative hypoPT (17). Temporal categorizations postsurgery included immediate versus delayed onset, further stratified by duration into transient and permanent conditions (**Table 1**).

**Table 1:** Distinct categories of postoperative hypoparathyroidism based on varying criteria.

Standard	Categorization	Definition
Diagnostic criteria	Biochemical hypoPT	Serum iPTH < 12 pg/ml*,potentially concurrent with hypocalcemia.
	Clinical hypoPT	Abnormal biochemical indicators with signs or symptoms of hypocalcemia
	Relative hypoPT/ Parathyroid insufficiency	Clinical manifestations of hypocalcemia with normal biochemical indicators
Occurrence time	Immediate hypoPT	Within 24 hours following surgery
	Delayed hypoPT	After 24-hour postsurgical window
	Transient hypoPT	Within 6 months following surgery
	Permanent hypoPT	Beyond 6 months postsurgery without recovery

iPTH, intact PTH. *Intact PTH levels below the lower limit of the laboratory reference range (typically 12 pg/ml).

## 4 Risk factors

In the United States, more than 130,000 thyroid surgeries are performed annually (18), and anterior neck surgeries are the most prevalent etiology of this condition, with acquired postoperative hypoPT accounting for approximately 75% of all cases. This encompasses mechanical trauma, thermal injury, surgery [total thyroidectomy (TT) vs. sub-total thyroidectomy (sub-TT) vs. lobectomy (LT)], neck dissection, intentional excision, inadvertent removal, and disruption of the vascular supply (19, 20). Moreover, technique and expertise of surgeon may significantly correlate with incidental parathyroidectomy. The remaining 25% cases of hypoPT may be attributed to genetic factors, autoimmune assaults, pseudohypoparathyroidism, and a minuscule percentage of infiltrative disease (21, 22).

**Figure 2:**
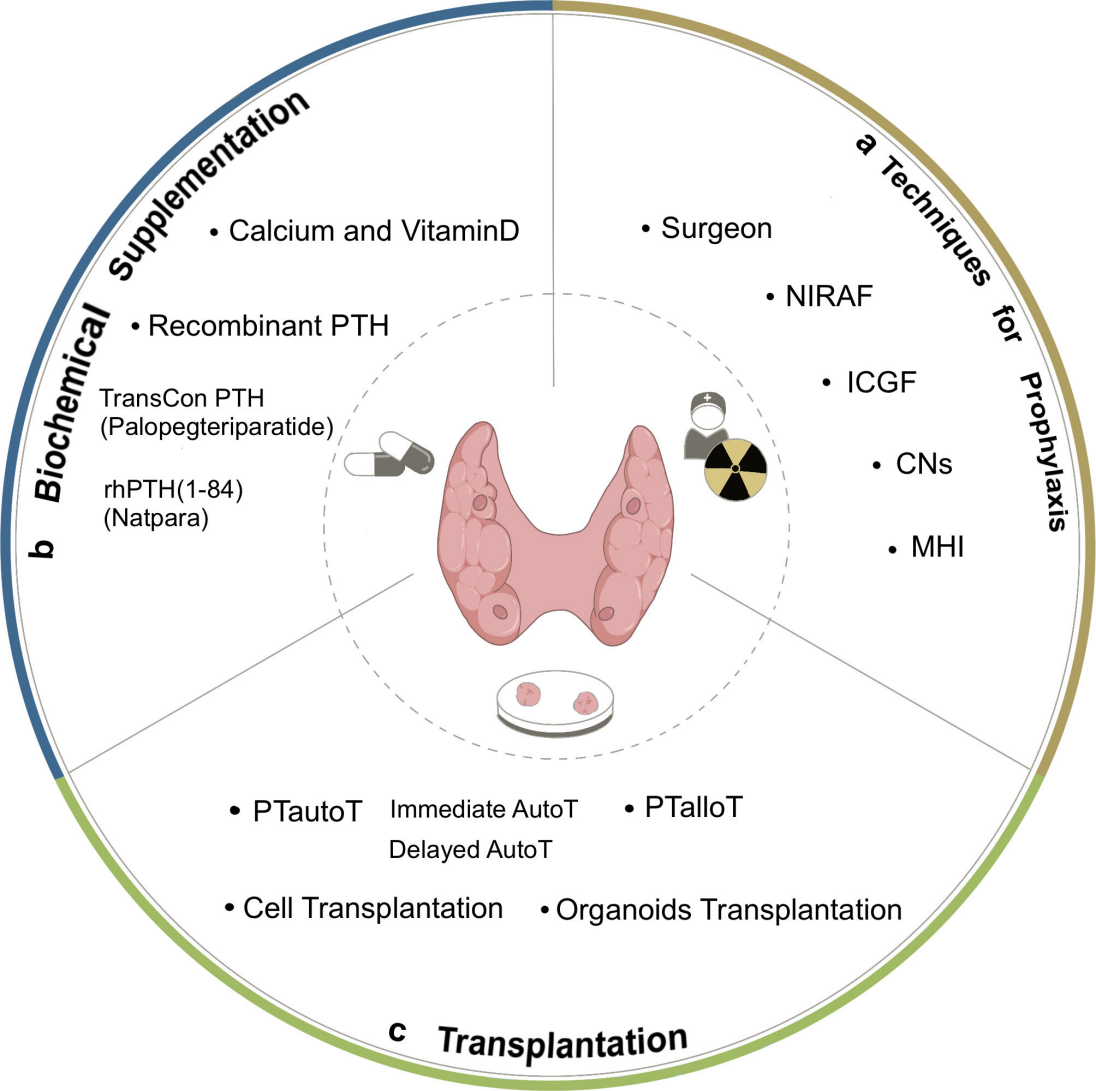
Options of prevention and treatment for post-operative hypoparathyroidism. **(a)** Techniques for prophylaxis. Experienced surgeons and use of auxiliary tools to reduce the hypoparathyroidism caused by surgery. NIRAF, near-infrared autofluorescence. ICGF, indocyanine green fluorescence. CNs, carbon nanoparticles. MHI, mitoxantrone hydrochloride. **(b)** Biochemical supplementation. Supplementation with calcium agents and vitamin D is crucial for treating hypoparathyroidism, Recombinant human PTH(1-84) (Natpara) is approved for adults refractory hypoPT in the US and Europe as a supplement. FDA approved TransCon PTH (Palopegteriparatide) as the first and only long-acting hormone replacement for hypoparathyroidism in adults. **(c)** Transplantation. Organ or cell transplantation represents a physiological option for managing endocrine disorders, PTautoT is commonly used in surgery, organoids emerge as a new option to replace damaged tissues. PTautoT, parathyroid autotransplantation. Immediate AutoT, immediate autotransplantation. Delayed AutoT, delayed autotransplantation. PTalloT, parathyroid allotransplantation.

HypoPT is also the most frequent complication following thyroid surgeries (23). The incidence of transient hypoPT ranges from 7.3% to 83%, and that of permanent hypoPT varies from 0% to 12.1% (24–28). With the annual increase in the number of thyroid surgeries, the prevalence is expected to increase. Importantly, once hypoPT manifests, its impact on patients could be profound and potentially lifelong. Therefore, prophylactic measures and therapeutic interventions for hypoPT are of utmost importance (**Figure 2**).

## 5 Techniques for prophylaxis

Prevention supersedes treatment for patients who undergo surgery. Intraoperative identification of fewer than two PGs is an independent predictor of permanent hypocalcemia following TT (25). Efficient identification, meticulous anatomy and *in situ* preservation of the glands and their vascular supply are the optimal approaches for ensuring intact parathyroid function (29).

### 5.1 Surgeon technique

The knowledge of normal anatomy and its variation is essential for surgeons. Approximately 85% of superior PGs are situated within a 1 cm radius around the inferior angle of the thyroid cartilage. Inferior PGs and the thymus, which originate from the third pharyngeal pouch, can migrate between the hyoid bone and upper mediastinum, making surgery difficult.

PGs primarily receive blood from two inferior thyroid artery (ITA) and two superior thyroid artery (STA), occasionally from a thyroid ima artery (TIMA) (30). TIMA is an uncommon anatomical variation, usually representing as compensatory for ITA absence (31). These terminal arterial branches lack collateral anastomoses, requiring careful dissection to preserve vascular supply, a safe distance of at least 3 mm from the vessels when energy-based instruments are used to prevent thermal damage, and the use of clamp and tie (CAT) technology to protect arterial branches (32, 33). Moreover, identification of the inferior parathyroid vein aids in preserving the inferior gland *in situ*. Nonetheless, even if the vascular anatomy remains intact during surgery, postoperative vascular spasms poses a risk for parathyroid dysfunction.

Consequently, owing to the inherent anatomical complexities of PGs, such as their diminutive dimensions (4–6 mm), delicate structure, coloration mimicking adjacent tissues, and variations in glandular location and vascularization, surgeons require extensive fundamental understanding and precise surgical technique. TT performed by high-volume surgeons lead to minimal endocrine-related complication rates about 1.6% and maximal cost effectiveness (34, 35).

### 5.2 Identification technique

Many surgeons rely on the empirical identification of PGs with naked eye (NE) based on anatomical location, and this subjective visual inspections are usually unreliable (36). Utilization of auxiliary technologies significantly improve the accuracy of parathyroid gland identification. The intraoperative application of Near-Infrared Autofluorescence (NIRAF), Indocyanine Green Fluorescence (ICGF), Carbon Nanoparticles (CNs), and Mitoxantrone Hydrochloride (MHI) facilitates the identification and *in situ* preservation of PGs, thereby reducing the incidence of postoperative complications (37). Therefore, it is imperative to evaluate the advantages and limitations of various methodologies.

#### 5.2.1 Near-infrared Autofluorescence

Under near-infrared light stimulation at wavelength of 760–770nm, PGs emit autofluorescence (AF) at 820–830nm (38, 39). Owing to the inherent cellular structure and low fat density signals, AF intensity of PGs is 2.4- to 8.5-fold stronger compared with the thyroid and background (40.6 ± 26.5 vs. 31.8 ± 22.3 vs. 16.6 ± 15.4). The difference in fluorescence intensity provides a reliable basis for intraoperative real-time identification of the PGs (40, 41).

NIRAF is a high accurate assurance of PGs. In 550 specimens, NIRAF signature is highly consistent with pathologic findings (sensitivity of 98%, specificity of 97%, positive predictive value of 95%, and negative predictive value of 99%) (42). NIRAF can detect an increased number of PGs than NE, probably because the penetration of near-infrared light is more than 3mm, with 98% of PGs could be detected AF, and 46% of PGs could be detectable even when obscured by soft tissue (38). NIRAF used during TT showed the best advantage in reducing the incidence of postoperative early hypocalcemia (from 20.9% to 5.2%), and reducing autotransplantation rate (43).

However, this technique also has certain limitations. NIRAF cannot assess the blood supply of PGs, and is incapable of guiding intraoperative decisions on autotransplantation. Furthermore, commercially available NIRAF devices involve complex operational procedures and lack high-quality consensus.

#### 5.2.2 Indocyanine Green Fluorescence

Indocyanine Green (ICG) is an amphiphilic tricarbocyanine dye rapidly binds to plasma lipoproteins after intravenous injection (44). ICG is taken up by the highly vascularized parathyroid glands, emitting fluorescence with a peak at 832 nm when excited by near-infrared light at wavelength of 800 nm (45). Furthermore, the varying fluorescence intensity positively correlates with the perfusion and functionality, facilitating intraoperative visual assessment of PGs functionlity and determining the *in situ* preservation. If at least one well vascularized PG is demonstrated by ICGF after surgery, the patient’s postoperative PTH levels could remain within the normal range without treatment (46).

The SUCRA values from a meta-analysis including 29 studies showed that the PGs identification rate of ICGF is significantly higher than that of NE (0.76 vs. 0.04) (47). However, ICGF have the highest autotransplantation rate. This is attributed to the fact that a lack of ICGF intensity—a potential indicator of impaired blood supply—inclined surgeons toward intraoperative autotransplantation. Consequently, the incidence of postoperative hypocalcemia and hypoPT in ICGF group showed no significant difference from that in NE group (47). ICGF has a lower PGs identification rate than NIRAF, as ICG absorption by thyroid glands impedes PGs visualization (44). A randomized controlled trial revealed combining AF with ICGF could reduce the risk of transient postoperative hypocalcemia, facilitate the identification and preservation of PGs, and improve the accuracy of intraoperative PG perfusion assessment (48).

Finally, it should be noted that ICG solution contains sodium iodide and is therefore contraindicated in patients with iodine allergy or renal impairment (49).

#### 5.2.3 Carbon Nanoparticles

CNs—a lymphatic tracing agent with 150 nm diameter—injected into the thyroid accumulate in lymph nodes during thyroidectomy, rapidly staining the thyroid gland and the lymph nodes black while preserving the anatomical color of the PGs and laryngeal nerves (50). According to SUCRA values, CNs have the best advantage in reducing the rate of transient postoperative hypoPT (0.95), followed by AF (0.67), ICGF (0.22), and NE (0.16) (47). Meanwhile, CNs are used for indirect identification of PGs. Compared to direct identification techniques (NIRAF, ICGF), CNs imaging exhibits an extremely high false-negative rate, with PGs identification accuracy only superior to visual inspection (47, 51).

Few side effects related to CNs have been reported. If the injection of CNs is too deep (penetrating the serosal layer) or the dose is excessive during surgery, it may cause dye extravasation. This could stain the surgical field and complicate the protection of the arteries supplying PGs, although this risk can be avoided with carefully administered during surgery (52).

#### 5.2.4 Mitoxantrone Hydrochloride

MHI—a novel lymph nodes tracer—was approved for the first time by the National Medical Products Administration (NMPA) for use in thyroidectomy in 2021. MHI self-assembles into nanocrystals (approximately 100 nm in diameter) in the interstitial spaces of thyroid through noncovalent interactions (53). The gap between capillary lymphatic endothelial cells is 100–500 nm while that between the capillary endothelial cells is only 30–50 nm (51). MHI nanocrystals cannot cross the capillary walls but can enter lymphatic capillaries. They accumulate in lymph nodes via lymphatic drainage and are captured by macrophages, thereby producing a blue tracer effect.

MHI detects PGs indirectly through lymph node negative imagin. Compared to NE, the use of MHI during TT enhances lymph nodes dissection and PGs identification, decreases the rate of inadvertent PGs resection, and reduces the incidence of transient postoperative hypoPT (14.29% vs. 32.5%) (54).

MHI is a safe and effective tracer. No adverse reactions associated with MHI have been observed, and the safe dosage range is 0.2–0.6 ml (55). Furthermore, MHI dye extravasation can be effectively removed by using gauze soaked in saline.

## 6 Therapeutic biochemical supplementation

The primary biochemical indicators for diagnosing hypoPT include abnormally low or undetectable serum levels of ionized PTH (iPTH), along with albumin-corrected total serum calcium [corrected calcium = total serum calcium measurement + 0.02 × (40 - albumin measurement)], since 40–45% of serum calcium is protein-bound calcium. Monitoring and ameliorating electrolyte imbalances are crucial for mitigating the risk of hypoPT.

### 6.1 Calcium and vitamin D supplementation

The half-life of PTH is approximately 3–5 minutes, and persistent PTH levels at or above 15 pg/ml for more than 20 minutes postoperatively indicate a low likelihood of postoperative PG dysfunction with no need for intensive serum calcium monitoring or supplementation (17). However, in the event of acute hypocalcemia, prompt calcium and active vitamin D supplementation can improve the chances of restoring PG functionality, reversing permanent dysfunction and keeping the patient alive (56).

Postoperative acute hypocalcemia typically manifests after serum PTH levels decrease, reaching their nadir within 24–72 hours. A serum calcium concentration less than 1.88 mmol/L within 24 hours is indicative of permanent hypocalcemia (57, 58). The “parathyroid splinting” hypothesis suggests that allowing damaged parathyroid tissue to rest after surgery, combined with pharmacological interventions to increase calcium levels, may improve long-term outcomes (59). The panel made a recommendation suggesting conventional therapy as first line therapy, including postoperative supplementation of calcium agents and active vitamin D, correction of abnormal serum magnesium.

#### 6.1.1 Calcium

Oral calcium agents, such as calcium carbonate and calcium citrate, are commonly used for supplementation. Calcium carbonate, which contains 40% elemental calcium, is preferred because of its cost-effectiveness and lower dosage requirements (60). However, its absorption depends on gastric acid, requiring lower doses of meals for those with normal gastric acid. Calcium citrate is an alternative for patients with specific conditions (undergone gastric bypass surgery, take acid-blocking medications, present bloating and constipation side effects of calcium carbonate, elders with reduced gastric acid), requiring approximately double the dose of calcium carbonate due to its 21% elemental calcium (61). Monitoring urinary calcium levels during supplementation is crucial to prevent the renal complication of calcium deposition. Typically, serum calcium levels should be within the lower normal range (8.0–8.5 mg/dl or 2.00–2.12 mmol/l), with urinary calcium levels under 300 mg/day (7.5 mmol/d). Moreover, oral calcium agents can inhibit intestinal levothyroxine absorption, so it is recommended that patients take levothyroxine either 1 hour before or 3 hours after calcium supplementation after thyroidectomy (62, 63). The intravenous administration of 10% calcium gluconate is effective in rapidly increasing blood calcium levels if oral supplements are ineffective, with careful monitoring of the patient’s electrocardiogram for the QT interval and any anomalies, as it poses significant risks especially in patients with cardiac complications. Therefore, it is always safe to offer infusions of 10% calcium gluconate diluted in 150 ml saline instead of direct intravenous administration which should be reserved for emergency conditions like laryngospasms.

#### 6.1.2 Vitamin D

Vitamin D maintains the calcium balance in the bloodstream by promoting calcium absorption in the intestines and bone resorption. There are various formulations of vitamin D, such as ergocalciferol (VD2), cholecalciferol (VD3), dihydrotachysterol, alfacalcidol (1α-OH-D3), and calcitriol [1,25-(OH)2-D3] (55). Calcitriol and alfacalcidol are bioactive forms of vitamin D that undergo 1α-hydroxylation, enhancing their potency by 100–500 times (64). They are commonly used in patients with hypoPT. Therefore, close monitoring of serum calcium levels is essential during drug therapy for hypoPT to prevent hypercalcemia and hypercalciuria caused by vitamin D toxicity (65). Additionally, their relatively short half-lives (4–6 hours) necessitate once- or twice-daily dosing, which improves patient compliance while facilitating timely correction of vitamin D toxicity (6).

However, a concern with vitamin D therapy is the risk of hyperphosphatemia, which can lead to the formation of insoluble calcium phosphate complexes in soft tissues such as the brain, blood vessels, and kidneys. Therefore, it is imperative to monitor phosphorus intake and electrolyte levels and maintain serum phosphate levels within the normal range or a calcium–phosphate product concentration ≤55 mg^2^/dl (1).

#### 6.1.3 Protocol

Supplementation may be prophylactically administered to patients at risk of postoperative PG dysfunction or on the basis of postoperative blood PTH and calcium level monitoring (**Table 2**) (66). These medications can assist patients in managing temporary PG dysfunction until normal serum calcium levels are stable. Gradual reduction in drug dosage, monitoring of biochemical markers, and avoidance of complications such as hypercalcemia are essential steps once PG function is restored.

**Table 2:** Pharmacological administrations for the treatment of hypoparathyroidism following surgery.

Protocol	Medication	Management in adults	Route
Experientially	Calcium carbonate	0.5-1.25 g of elemental calcium, divided into 2-3 doses	Oral
	Calcitriol	0.25-0.5 μg, divided into 2 doses	Oral
PTH<15pg/ml, serum calcium<8.5mg/dl (2.12mmol/l) /i-onized calcium<1.1mmol/l	Calcium carbonate	1-3 g of elemental calcium	Oral
	Calcium citrate	2-6 g (400-1200 mg of elemental calcium)	Oral
Maintained serum calcium<7mg/dl (1.75mmol/l) /manife-stations of hypocalcemia	Calcium carbonate	1-3 g of elemental calcium	Oral
	Calcium citrate	2-6 g (400-1200 mg of elemental calcium)	Oral
	Calcitriol	0.25-0.5 μg, divided into 2 doses .	Oral
severe hypocalcemia despite oral calcium and calcitriol	Calcium gluconate 10%	10-20 ml (93-186 mg of elemental calcium) over 10-15 min	I.V.
		1.25 mg of elemental calcium per kilogram q.h.	I.V.gtt
Refractory hypoparathyroidism	PTH(1-84) (Natpara) TransCon PTH(Palopegteriparatide)	50-100 μg q.d. initial dose of 18 μg q.d. increase palopegteriparatide by 3 μg every 7 days	S.C. S.C.

The inability to recover PG function within six months postsurgery indicates permanent dysfunction, requiring lifelong management to maintain normal serum calcium levels, prevent multiorgan complications, and increase patients’ quality of life. Conventional pharmacotherapy can control serum calcium levels but does not correct the underlying PTH deficiency. This deficiency can lead to physiological disturbances, including reduced lomerular filtered of calcium, altered renal calcium reabsorption, and decrease bone turnover.

Thiazide diuretics, along with calcium supplements, can prevent excessive calcium loss (67–69). However, thiazides may cause magnesium loss (<1.6 mg/dl), affecting PTH release, and oral magnesium oxide 400 mg, once or twice daily, is recommended (70). PTH deficiency also affects magnesium reabsorption in the distal convoluted tubules. Concurrently, magnesium deficiency leads to PTH resistance, as magnesium serves as a cofactor for adenylate cyclase. During conventional treatment, patients with hypomagnesemia should receive magnesium supplements to restore normal plasma magnesium levels (71). Additionally, elevated serum magnesium levels reduce the synthesis and secretion of PTH by binding to CaSR, necessitating close monitoring (71).

### 6.2 Recombinant PTH

HypoPT presents a unique challenge in endocrine deficiency disorders, as hormone replacement therapy is avoided previously because of concerns over osteosarcoma development in rat studies (72, 73). However, human trials have not shown this risk. The FDA and EU approved rhPTH(1-84) (Natpara) for adults with refractory hypoPT in the US and Europe as a supplement to conventional therapies that are poorly controlled, require high-dose medications, or severely impact physical and mental health. Recombinant human PTH(1-34) (Teriparatide) is approved for the treatment of osteoporosis, and clinical trials on hypoPT with rhPTH (50–100 μg/day) have been effective in stabilizing renal function, increasing bone turnover, and reducing the use of oral supplements (0 μg/day active vitamin D and ≤500 mg/day calcium) (74). Consequently, in Augest 12, 2024, FDA approved TransCon PTH (Palopegteriparatide, approved under the brand name YORVIPATH^®^ in the EU) as the first and only long-acting hormone replacement for hypoparathyroidism in adults, based on the phase 3 PaTHway trial and phase 2 PaTH Forward trial. This PTH analog is a prodrug of teriparatide that releases PTH(1–34) from carrier through autocleavage of the TransCon linker, administered once-daily subcutaneous injection, with 60-hour apparent half-life and 24-hour sustained release of active PTH (75). In contrast, natpara has a short half-life of 3 hours, once-daily injection leads to wide PTH fluctuations (76). The efficacy is also a critical differences between palopegteriparatide and natpara. In PaTHway, 90% of patients receiving palopegteriparatide independent from conventional therapy by week 26 (77). In contrast with the REPLACE trial, approximately 40% of patients receiving natpara achieved independence (78).

Over time, patients with chronic hypoparathyroidism or managed with conventional therapy can increase the risk of chronic renal complications including nephrocalcinosis and nephrolithiasis. Nevertheless, the effect of natpara on renal calcium remains controversial. It transiently increases the glomerular filtration rate (10–12 h), promoting renal calcium reabsorption and reducing urinary calcium excretion, thereby increasing the risk of long-term renal complications and nephrocalcinosis (79). However, palopegteriparatide treatment is beneficial to individuals, may preserve or improve renal function as observed in trial with a mean increase in estimated glomerrular filtration rate of 9.3ml/min/1.73 m^2^ (80). Nonetheless, there are adverse reactions related to palopegteriparatide during the 26-week trial with an incidence ≥5% (injection site reaction, headache, unintended changes in calcium levels, orthostatic hypotension, digoxin toxicity with concomitant use of digitalis compounds, etc.), and limited assessments of osteosarcoma beyond 2 years with no recommendation in patients at risk of osteosarcoma. Additionally, this treatment is costly, and further observational studies are needed for widespread application due to the limited clinical data on long-term safety and efficacy.

## 7 Transplantation for therapy

For patients with permanent hypoPT, while oral calcium supplements or hormone replacement can compensate for endocrine imbalances, they cannot replicate the complex metabolic interactions of the hormone. Moreover, long-term pharmacotherapy involves various complications, imposing a significant medical burden on patients and physicians. Organ or cell transplantation represents a physiological option for managing endocrine disorders.

### 7.1 Parathyroid autotransplantation

Parathyroid autotransplantation (PTautoT) can significantly reduce the incidence of permanent hypoPT by salvaging unintentionally resected or blood-cessated glands, with success rates ranging from 75% to 100% (81–84). It is supported by the ability of parathyroid tissue to increase vascular endothelial growth factor expression, inducing angiogenesis (85). Vascular growth and functional recovery of the transplanted tissue occur within 10 days to 12 months (86–88).

#### 7.1.1 Immediate autotransplantation

Immediate autotransplantation involves a technique established by Wells et al. (83, 89) The tissue is placed in 4 °C saline or culture medium and sliced into 1*1*2 mm pieces after rapid PTH assays, frozen section pathology and a 30-minute cooling period. The specimens are transplanted into disperse muscle pockets and then closed with metal clips or nonabsorbable sutures. The brachioradialis muscle of the nondominant arm is commonly selected for transplantation because of its abundant blood supply and convenience for subsequent evaluation. The sternocleidomastoid muscle, pectoralis major, thigh muscles, and trapezius on the same side were also utilized. However, the recent meta-analysis and trial sequential analysis showed that PTautoT in the sternocleidomastoid muscle is not associated with a decreased risk of permanent hypoPT, while PTautoT in the brachioradialis muscle does decrease the incidence of permanent hypoPT (90).

Serum PTH levels progressively increase, maintain normal levels by the fourth week, and sustain stabilization for six months without additional supplementation after transplantation (22, 91). Compared with at least one gland autotransplantation without PTH assessment, selective parathyroid autotransplantation with intraoperative PTH <10 ng/l successfully prevents permanent hypoPT and significantly reduces the risk of transient hypoPT (92).

#### 7.1.2 Delayed autotransplantation

Delayed autotransplantation refers to preserving the tissue in liquid nitrogen with 10% dimethyl sulfoxide as a cryoprotectant until six months postsurgery, and there is still no parathyroid function (22, 93). However, tissues are not recommended to freeze for more than two years, as tissue viability decreases over time; for example, the transplantation success rate is only 80% at nine months in rat trials (83).

#### 7.1.3 Indications

*In situ* identification and preservation of PGs is the optimal strategy to prevent postoperative hypoPT. PTautoT is also a commonly used strategy for preventing and treating postoperative hypoPT. For PGs identified intraoperatively that cannot be preserved *in situ*, immediate autotransplantation should be performed. For example, immediate autotransplantation should be performed proactively in the following scenarios: - Intraoperative observation of pale PGs indicating complete compromised blood supply - Unintended resection of PGs identified in thyroid, central lymph node, or adipose tissue specimens - Cases where thyroid cancer is tightly adherent to PGs, precluding their preservation - High-risk surgeries (advanced cancer, reoperations) where *in situ* preservation of all PGs is anticipated to be challenging (82, 94). For extremely high-risk patients, non-preservable PG tissue should be cryopreserved for delayed autotransplantation in case of permanent hypoPT. For example, in cases involving bilateral central zone lymph node dissection or reoperation, multiple (≥2) PGs may be resected or suffer vascular compromise (95). Even if immediate autotransplantation has been performed, cryopreservation may be considered if the number of *in situ* preserved PGs is extremely limited (≤1) and their function is uncertain (96).

However, some reports refute the effectiveness of autotransplantation in preventing parathyroid dysfunction, as no significant differences were observed in clinical symptoms or calcium and iPTH levels during follow-up, regardless of whether the parathyroid implant was used (97, 98). Because fewer than four glands are retained *in situ* and the viability is doubted (99–101), the incidence of temporary hypoPT increases with the number of transplants (102–104). A meta-analysis of 18 studies indicated that PTautoT is associated with transient hypoPT but has no effect on the incidence of permanent hypoPT (90).

### 7.2 Parathyroid allotransplantation

Allotransplantation is a potential therapeutic modality for which HLA matching may not be necessary (105). The transplants can come from brain-dead donors, individuals with hyperparathyroidism (transplanting adenomas might induce hyperparathyroidism), or parathyroid-like cells derived from human embryonic stem cells, which are cultured and assessed for viability and functionality (105). Most recipients neither exhibit hypocalcemia nor require long-term immunosuppression (106, 107). However, the survival rate of allografts within 12 months is less than 50%, with the longest reported survival being only three years, necessitating multiple retransplantations (105). Owing to uncertainties about immunosuppressant application, complications, and limited long-term efficacy, further research is needed before widespread clinical application.

### 7.3 Parathyroid cell transplantation

Parathyroid cells cultured *in vitro* can downregulate MHC-I and MHC-II expression, reducing immunogenicity (108). This process can also control the quantity of implanted cells, regulate the PTH content, cryopreserve and reuse them (108, 109). Proliferative and PTH-secreting cells can be obtained via cell culture passaging, then, alone or in microcapsules, can be implanted into the muscle, abdominal wall, or peritoneum intravenously or subcutaneously, leading to increased serum PTH and calcium concentrations (110). However, cell transplantation has issues such as limited cell duration posttransplantation, restricted cell quantities at transplant sites, rejection in allogeneic cell transplants, and challenges with microencapsulation technology (111, 112). Consequently, the effectiveness of clinical application requires further evaluation.

## 8 Transplantation of organoids

Organoids are self-organizing assemblies that feature specific spatial structures and functions that closely resemble the physiological and pathological profiles of human organs; they are formed through *in vitro* three-dimensional cultures of somatic cells, adult stem cells (including progenitor cells), or pluripotent stem cells (113). Due to their significant advantages over conventional cell and animal models in terms of physiological processes, genetic stability, and ease of operation, organoid technology has presents a novel approach for medical research and is rapidly being implemented in studies on organ development, disease modeling, drug screening, toxicity testing, precision medicine, and regenerative medicine (114–117).

### 8.1 Organoid transplantation

Organoids serve as reserves for transplantation to replace or repair damaged tissues. Researchers have successfully transplanted retinal pigment epithelial cells into patients with age-related macular degeneration. Moreover, liver organoids transplanted into mice integrate functionally within two days and reverse liver failure (118–120). Transplanting intestinal epithelial organoids into a mouse model of ulcerative colitis validated the use of transplantation therapy for refractory ulcerative colitis (121). Thyroid organoids are constructed from induced pluripotent stem cells, which directly simulate key events in development and demonstrate the ability to secrete hormones posttransplantation into mice, advancing regenerative therapies (122).

### 8.2 Parathyroid organoids

Despite early reports of human embryonic stem cells differentiating into parathyroid-like tissues via activin A and Shh proteins (123, 124), research on parathyroid organoids has long been limited because of their small tissue volume, scarce cell numbers, and difficulty in sample acquisition (125). However, a recent breakthrough at the University Medical Center Groningen involved the isolation of stem cells from patients with benign parathyroid hyperplasia to construct parathyroid organoids (126). As demonstrated through hormone secretion monitoring, drug response testing, tracer experiments, and near-infrared autofluorescence properties, these organoids are highly appropriate structured models capable of mimicking functional parathyroid tissues (127). Other researchers have also cultured organoids with intact secretory function from the tissues of 6 patients with benign primary hyperparathyroidism, elucidating the feasibility of using organoids in disease and transplantation research through bioenergetic and metabolic analyses (128). Recently, parathyroid organoids have successfully differentiated from human embryonic stem cells, resulting in an increased differentiation rate and PTH secretion through the overexpression of the GCM2 protein (129). Additionally, researchers generated parathyroid organoids using fibroblast-derived induced pluripotent stem cells and characterized their functional activity and tissue integration potential through *in vitro* validation and *in vivo* experiments involving rat transplantation (130). This strategy avoids the ethical concerns associated with embryonic stem cells. Therefore, advanced organoid technology has immense potential for regeneration and offers a viable therapeutic option for treating permanent hypoPT. However, challenges and barriers exist with organoid replacement therapy, including cell diversity, the cultivation environment, the duration of functional maintenance, and immunological rejection.

## 9 Conclusion

Hypoparathyroidism remains one of the most common postoperative complications following thyroid surgery. Strategies such as enhancing surgical expertise, perioperative monitoring, supplementation with PTH or calcium and vitamin D, fluorescence technologies, contrast agents, autotransplantation or allotransplantation have been employed for its prevention and treatment. However, the current methodologies are insufficient for completely curing permanent hypoPT, which, once established, impairs quality of life indefinitely. This reality underscores the clinical imperative for a novel, effective treatment modality to manage intractable hypoPT cases, with organoid transplantation emerging as a promising frontier in the quest to address hypoparathyroidism. Palopegteriparatide has been approved for use in patients with hypoparathyroidism as a replacement for PTH therapy. Tracking its long-term clinical efficacy, adverse reactions, and economic impact would be meaningful.
